# Androgens and Hirsutism in a Large Cohort of Portuguese Women

**DOI:** 10.3390/jcm14030673

**Published:** 2025-01-21

**Authors:** Joana Pinto, Nicoletta Cera, Claudia Camerino, Jorge Beires, Duarte Pignatelli

**Affiliations:** 1 Faculty of Medicine, University of Porto, 4200-319 Porto, Portugal; up201707095@up.pt; 2 Faculty of Psychology and Education Sciences, University of Porto, 4200-135 Porto, Portugal; 3 Research Unit in Medical Imaging and Radiotherapy, Cross I&D Lisbon Research Centre, Escola Superior de Saúde da Cruz Vermelha Portuguesa, 1300-125 Lisbon, Portugal; 4 Department of Precision and Regenerative Medicine, School of Medicine, University of Bari Aldo Moro, P.za G. Cesare 11, 70100 Bari, Italy; ccamerino@libero.it; 5 Department of Obstetrics and Gynecology, Centro Hospitalar e Universitário de S. João, 4200-319 Porto, Portugal; 6 Department of Endocrinology, Centro Hospitalar Universitário de São João, 4200-319 Porto, Portugal

**Keywords:** androgens, hirsutism, polycystic ovary syndrome, non-classic congenital adrenal hyperplasia, idiopathic hyperandrogenemia, idiopathic hirsutism, obesity

## Abstract

**Background/Objectives**: Hirsutism is excessive male-patterned hair in postpubertal women with multifactorial etiology and is an indicator of hyperandrogenism associated with polycystic ovary syndrome (PCOS). Indeed, it can be caused by the enhanced peripheral conversion of androgen precursors to testosterone, as in idiopathic hirsutism (IH). Moreover, hirsutism can be caused by hirsutism-related hyperandrogenic syndromes like non-classic congenital adrenal hyperplasia (NCAH) and idiopathic hyperandrogenism (IHA). **Methods**: In this study, we characterized a large cohort of Portuguese women referred for hirsutism and estimated the prevalence of PCOS, NCAH, IHA, and IH. The levels of androgens and gonadotropins and body mass index (BMI) were measured and compared with controls. The correlation between each variable was calculated. **Results**: In the cohort, we found a prevalence of PCOS of 56.2%, IH of 20.2%, IHA of 17.3%, and NCAH of 6.2%. Subjects with PCOS were the only ones showing a significant difference in BMI compared to the controls and had the lowest levels of sex hormone-binding globulin (SHBG). Those with NCAH were younger and more hirsute with higher levels of testosterone, among other androgens. Those with IH had lower luteinizing hormone (LH) and LH/follicle-stimulating hormone (FSH) ratios than those with PCOS. Those with IH had lower SHBG levels compared to the controls and a higher free androgen index (FAI). Those with IHA had higher androgens compared to those with IH, in particular, adrenal-derived androgens. **Conclusions**: The pathogenesis of hirsutism is complex, and the contributions of the pituitary gland, ovaries, adrenals, adipose tissue, and liver have to be ascertained to understand the clinical manifestations and delineate appropriate treatments. This study sheds new light on the fine hormonal regulation of these diseases.

## 1. Introduction

Hirsutism refers to the presence of excessive male-pattern terminal hair in women after puberty, affecting facial and other androgenic-dependent areas [1]. It is a recurrent reason for dermatological and endocrinological consultations [2] since it impacts 5% to 15% of premenopausal women worldwide [3] and is correlated with a decreased quality of life in women [4].

Hirsutism have a multifactorial etiology, as we will explain below. Indeed, hirsutism is often caused by high levels of androgens [5] secreted from the ovaries/adrenal glands or an increased sensitivity of hair follicles to normal androgen levels. Adrenal androgen production is dependent on adrenocorticotropic hormone (ACTH), while luteinizing hormone (LH) regulates ovarian androgen production [6,7].

Hirsutism can also be caused by the enhanced peripheral metabolization of steroid precursors and enhanced androgen levels. For instance, just an estimated 33% of circulating testosterone (T) is produced by the theca cells of the ovaries [8], and the remaining T is derived from androstenedione (A4). The precursor A4 is produced by both the ovaries and adrenal glands and is then converted to testosterone in peripheral tissues [7]. Then, in granulosa cells of the ovary and peripheral tissues, testosterone is converted to dihydrotestosterone (DHT) by 5α-reductase, amplifying its effect on androgen-sensitive tissues [7,9,10]. Moreover, DHT interacts with androgen receptors (AR) in susceptible hair follicle cells, triggering the transcription of genes responsible for keratinocyte proliferation, the enhanced vascularization of follicles, and the development of terminal hair [11]. Even non-metabolized androgens can directly activate ARs when present at sufficient concentrations [12]. Nevertheless, hair follicles not only respond to androgens by widely expressing androgen receptors, but they also contain androgen-metabolizing enzymes, which are crucial in regulating androgen levels within the follicle [13,14,15,16].

Hence, the severity of hirsutism is frequently not correlated with the levels of circulating androgens even though measurable hyperandrogenemia has been detected in 80–90% of women with hirsutism [17]. A well-known example of this condition is idiopathic hirsutism (IH), which is defined by the presence of hirsutism without any circulating hormonal imbalances or gynecological features [5,18]. Its prevalence can vary from 5% up to 20% of hirsutism cases [18,19,20], with a higher manifestation rate in Mediterranean, Middle Eastern, and South Asian ethnicities [9].

In addition to ethnicity, skin type might also be relevant to hirsutism evaluation. A recent study evaluated hirsutism in 341 women using the Fitzpatrick classification, classifying skin type according to its susceptibility to sunburn and melanin production in response to sunlight [21]. They found a positive correlation between hirsutism, Fitzpatrick skin type (FST), and ethnicity, with an increasing prevalence of hirsutism in the group of patients with higher FSTs and with Hispanic, Middle Eastern, African American, and South Asian ethnicities. Interestingly, 276 participants of the study were diagnosed with polycystic ovary syndrome (PCOS), which is the most common cause of hirsutism, accounting for three out of every four cases [22].

Despite all of this, hirsutism is a reliable cutaneous indicator of hyperandrogenism, which is most frequently associated with PCOS [6,21,23].

PCOS is the most prevalent multifactorial endocrine disorder in women of reproductive age [24] and is associated with clinical or biochemical androgen excess, ovulatory dysfunction, and polycystic ovarian morphology (PCOM) [25,26]. This condition negatively impacts patients’ quality of life, not only because of hirsutism but also due to its gynecologic alterations and infertility [27,28], not forgetting its correlation with several metabolic, cardiovascular, and psychiatric comorbidities [29,30].

Despite the majority of hirsute women having either PCOS or IH, there are other rarer hirsutism-related hyperandrogenic syndromes, such as non-classic congenital adrenal hyperplasia (NCAH) [31,32] and idiopathic hyperandrogenemia (IHA), not to mention virilizing tumors of the adrenals and ovaries, which are even rarer.

NCAH is an autosomal recessive disorder caused by a partial deficiency of one of the enzymes involved in adrenal steroid synthesis, in particular, 21-hydroxylase, resulting in a less severe phenotype of CAH [33] clinically similar to PCOS [32]. NCAH is the most prevalent autosomal recessive genetic disorder in humans [34], and a relatively common disease [35], accounting for 1–10% of hyperandrogenic women [36,37]. It shows some ethnic variations, being more prevalent in the Mediterranean, Middle Eastern Ashkenazi Jewish, and Indian populations [32,38]. This dysfunction is characterized by elevated adrenal androgen production, which results in a disorder that is clinically difficult to distinguish from PCOS because it secondarily affects ovarian androgen production and can have similar ovarian polycystic morphology [37]. It typically manifests in childhood or early adulthood. Differentiating between NCAH and PCOS is crucial for effective management, as these disorders require distinct treatment approaches.

Contrary to PCOS, IHA is defined by the presence of clinical together with biochemical hyperandrogenism in women without ovulatory dysfunction or PCOM [39]. Women with IHA may present with hirsutism, acne, or androgenic alopecia while maintaining regular menstrual cycles. It has been reported that IHA accounts for approximately 15% of hirsutism cases [5].

The pathophysiology of IHA is not fully understood, but it is believed to involve increased androgen production together with heightened androgen receptor sensitivity. Despite elevated serum androgen levels, there are no ovarian abnormalities, thus distinguishing IHA from conditions such as PCOS. In the clinical setting, the diagnostic distinction between these conditions may be difficult and time-consuming, but of extreme importance, as different management, treatments, and follow-up may be needed, and condition outcomes, as well as the well-being of patients, may differ significantly [32,40].

Besides one study showing a high prevalence of 7% of hirsutism in a cohort of unselected Spanish women [41], no studies in the Portuguese population have been performed to characterize this type of patient. Our primary aim was to estimate the prevalence of PCOS, NCAH, IHA, and IH, the diseases primarily associated with hirsutism, in a cohort of women referred for hirsutism to an endocrinology consultation at a tertiary care medical center. Given the wide variety of pathophysiologies amongst different hirsutism-associated syndromes, we also aimed to characterize the patients biochemically, correlating their hirsutism score to androgen levels and gonadotropins, among other indexes.

Finally, considering that hirsutism has also been associated with metabolic dysfunction [42], our secondary aim was to understand the differences between the mentioned hirsutism-related syndromes and body mass index (BMI).

We calculated the correlation between each variable analyzed for all four groups and calculated the discrimination power of the variables to differentiate each group.

## 2. Materials and Methods

### 2.1. Study Design

We conducted a cross-sectional study using data from 600 consecutive women attending endocrinology consultations between 2010 and 2022 at the University Hospital “São João” of Porto, a tertiary care academic medical center. The local ethics committee approved the study (Prot.n.23000471).

### 2.2. Participants

All hirsute women who attended endocrinology consultations at CHUSJ between 2010 and 2022, referred by general practitioners or other medical specialists, were comprehensively studied using the same protocol. Consequently, all the patients who had attended consultations for hirsutism were included.

Patients were included in the study cohort if they were women with an age of onset ≥ 16 years with documented clinical hirsutism. The exclusion criteria were as follows: (1) patients on oral combined contraceptives or other hormonal treatments; (2) women at menopause (defined as not having menstruated for at least 12 consecutive months after reaching 45 years of age); (3) women with a diagnosis of thyroid abnormalities, hyperprolactinemia, Cushing’s syndrome, and ovarian or adrenal tumors. To avoid sample bias, consecutive patients were recruited.

The controls were recruited among women of approximately the same age who were not taking oral combined contraceptives or other hormonal treatments and without hirsutism, irregular menses, or polycystic ovaries.

We evaluated the hirsutism scores and anthropometric variables in every patient and the controls. The hormonal parameters that were considered in this study population included total testosterone (TT-ng/mL), free testosterone (FT-pg/mL), androstenedione (A4 -ng/mL), dehydroepiandrosterone sulfate (DHEAS-ng/mL), sex hormone-binding globulin (SHBG-nmol/L), luteinizing hormone (LH-mIU/mL), follicle-stimulating hormone (FSH-mIU/mL), LH-FSH ratio, and 17-hydroxyprogesterone basal (17-OHP-ng/mL), which were determined using a Chemiluminescence Immunoassay. If 17-OHP was below 10 ng/mL, every woman undertook a Synacthen Test to exclude the presence of NCAH. In case of the occurrence of a positive result, genotyping of *CYP21A2* was performed to confirm it. These analytical data were collected in the first 3–7 days of the menstrual cycle. Estradiol and progesterone were determined at the same follicular phase, and at a presumptive luteal phase as well, in women with more-or-less regular cyclicity. Moreover, the modified Ferriman–Gallwey (mFG) score and the free-androgen index (FAI) were also calculated.

The definition of PCOS, NCAH, IHA, and IH followed the commonly described diagnostic criteria. A PCOS diagnosis was considered when women displayed 2 out of 3 of the following: (1) hirsutism with an mFG score of ≥ 8 or hyperandrogenemia with androgens above the upper normal limit (total T ≥ 0.73 ng/mL, free T ≥ 2.5 pg/mL, androstenedione ≥ 3.1 ng/mL, or DHEAS ≥ 4300 ng/mL); (2) oligo-anovulation with <9 cycles/year or, if cycles were regular, confirmed by low progesterone levels in the expected luteal phase of the cycle (confirmed twice); (3) PCOM that was defined as a follicular number of ≥12 per ovary, or an ovary volume ≥ 10 mL in at least one ovary. NCAH was diagnosed when 17-OHP was ≥ 10 ng/mL 0, 30, or 60 min after tetracosactide stimulation (short Synacthen test), regardless of other signs. The IHA group included hyperandrogenic patients with normal ovulatory cycles and normal ovarian morphology but with elevated androgen blood levels [43]. IH was defined as hirsutism with an mFG score ≥ 8 in the absence of hyperandrogenemia, menstrual dysfunction, or PCOM.

### 2.3. Data Analysis

All the collected data were entered into an Excel spreadsheet (Microsoft, Seattle, WA, USA), and all the statistical analyses were performed using Jamovi (Version 2.5-The Jamovi project, 2024—retrieved from https://www.jamovi.org). The prevalence of PCOS, NCAH, IHA, and IH was calculated. A Kolmogorov–Smirnov test was used to determine the distribution of the variables for each of the groups taken into consideration. All variables followed a non-normal distribution (*p* < 0.05) in our study population, and consequently, the data are presented as medians and interquartile ranges. A Kruskal–Wallis test was used to perform the between-group comparisons. After that, we calculated a series of Dwass–Steel–Critchlow–Fligner (DSCF) tests [44] for multiple-comparison analyses of the differences between the median values of the above-mentioned variables. A Bonferroni correction was then applied to all the obtained results. Spearman’s rank correlation coefficient was used to evaluate the relationships between the variables in each group, considering a 95% confidence level. Moreover, to assess the discrimination power of each index, we performed a series of receiver operating characteristic (ROC) analyses.

## 3. Results

### 3.1. Description of Population

**Figure 1:**
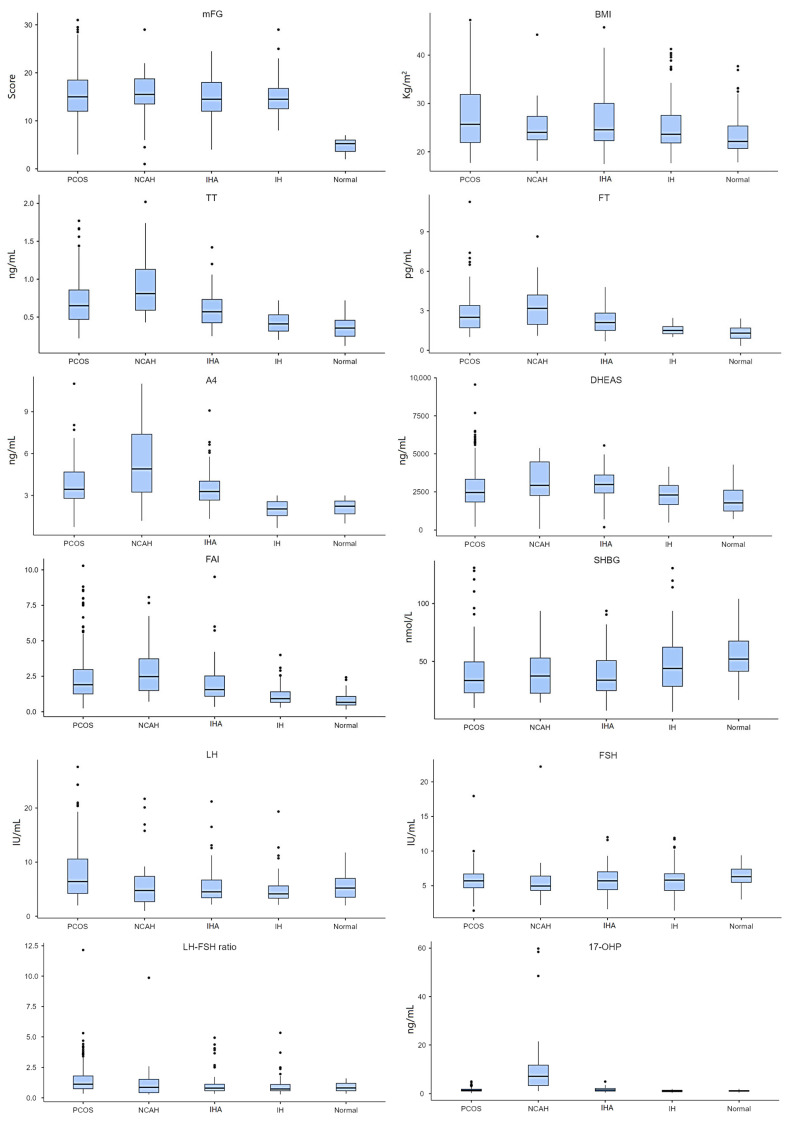
Between-group results.

A total of 564 cases were included. The remaining participants were excluded due to not having completed the study in its entirety. Controls represented 8.9% of the cohort. Overall, 91.1% of the subjects in the study sample had one of the four main diagnoses associated with hirsutism. Excluding the control group and considering only the patients who presented with hirsutism-related syndromes (*n* = 514), the prevalence of PCOS was 56.2%, of NCAH was 6.2%, of IHA was 17.3%, and of IH was 20.2% of the total patient group. The number of cases above the clinical cut-offs defined for each parameter was analyzed (Table 1), and the demographic data of the study participants are depicted in Table 2 and Figure 1.

### 3.2. Biochemical Differences Between PCOS, NCAH, IHA, and IH and Control Groups

In our study sample, patients from all the groups were slightly, but significantly, younger than the controls, with a *p*-value < 0.001 for PCOS, IHA, and NCAH, and *p* = 0.011 (unc.) for IH (Table 2).

Concerning hirsutism and androgen levels, PCOS patients presented significantly higher mFG scores and DHEAS levels than the controls (*p* < 0.001). When compared with the IH and control groups, both PCOS and NCAH patients presented considerably higher levels of TT (*p* < 0.001), FT (*p* < 0.001), and A4 (*p* < 0.001) as well as greater FAI scores (*p* < 0.001), respectively. Concomitantly, SHBG levels were significantly lower in PCOS patients than in IH patients (*p* = 0.003) and the controls (*p* < 0.001).

Curiously, PCOS patients showed lower values than NCAH patients in TT (*p* = 0.02 unc.) and A4 (*p* = 0.009 unc.). Furthermore, DHEAS showed lower values in PCOS patients than IHA patients (*p* = 0.007 unc.). NCAH patients presented higher levels than IHA patients of TT (*p* < 0.001), FT (*p* = 0.017 unc.), and A4 (*p* = 0.003). Additionally, DHEAS levels were higher in NCAH patients than in IH patients (*p* = 0.013 unc.) and the controls (*p* = 0.001). IHA patients had higher levels of TT (*p* < 0.001), FT (*p* < 0.001), A4 (*p* < 0.001), DHEAS (*p* < 0.001), and FAI (*p* < 0.001) than the IH and control groups.

Regarding SHBG, NCAH patients showed lower levels than the controls (*p* = 0.019 unc.). Moreover, IHA patients also had significantly higher values of mFG (*p* < 0.001) and lower SHBG levels (*p* < 0.001) than the controls. Likewise, in the IH group, we observed higher levels of mFG (*p* < 0.001), without increased androgen levels, but curiously, with increased FAI (*p* = 0.014 unc.) compared to the Control group. This probably reflects the lower SHBG levels than in the controls.

Concerning gonadotropin and progesterone levels, PCOS patients had higher levels of LH compared to the IHA (*p* < 0.001), IH (*p* < 0.001), and control groups (*p* = 0.027 unc.). Furthermore, the levels of the LH-FSH ratio and 17-OHP were higher in PCOS patients than in the IH (*p* < 0.001) and control groups (*p* < 0.001). Compared to IHA patients, PCOS patients also showed higher LH-FSH ratio values (*p* < 0.001).

As expected, PCOS patients showed lower 17-OHP levels than NCAH patients (*p* < 0.001). In addition, NCAH patients presented lower levels of FSH than the controls (*p* = 0.031 unc.) and higher values of 17-OHP than the IHA (*p* < 0.001), IH (*p* < 0.001), and control groups (*p* < 0.001). Compared to the IH and control groups, IHA patients also had higher levels of 17-OHP (*p* < 0.001).

Only PCOS patients showed significantly higher BMI scores than the controls, in terms of metabolic parameters. No other differences in BMI were found (Table 2).

To assess the effect of BMI, as distributed across the groups, on the biochemical parameters, we performed a series of nonparametric covariance analyses using the Quade test. BMI had a significant effect on all the parameters (*p* < 0.001, and *p* = 0.031 for FSH).

Pairwise comparisons of the groups for mFG showed a significant effect for the comparison between PCOS, NCAH, IHA, IH, and controls (*p* < 0.001). All the between-group comparisons for TT were significant (*p* < 0.001 and *p* = 0.026 for PCOS vs. IHA) except for IH vs. controls (*p* = 0.184).

A similar result (*p* < 0.001) was shown for FT, except PCOS vs. NCAH (*p* = 0.026) and vs. IHA (*p* = 0.035), for A4 (*p* < 0.001), with a non-significant BMI effect for PCOS vs. NCAH (*p* = 0.3) and IH vs. the controls (*p* = 0.3), and FAI.

DHEAS showed a significant BMI effect for all the comparisons (*p* < 0.001), except for NCAH vs. IHA and IH vs. the controls.

SHBG, with a *p* < 0.001 effect for BMI, showed non-significant results for PCOS vs. NCAH (*p* = 0.73) and IHA (*p* = 0.668).

17-OHP showed a non-significant effect for the comparison between PCOS and IHA (*p* = 0.131) and IH vs. the controls (*p* = 0.994).

For FSH, we observed a significant effect only for the different patient groups compared to the controls (*p* > 0.05), while LH and the LH-FSH ratio showed a significant effect for the comparison of the PCOS group with all the other groups (*p* < 0.002).

**Figure 2:**
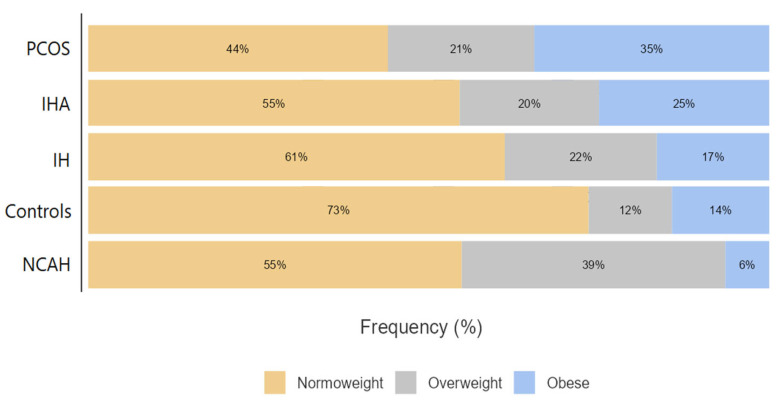
Stacked-bar plot indicating the prevalence of normoweight, overweight, and obese for each group.

Regarding BMI’s significance in most of the other parameters, the groups were further divided into BMI subgroups, and their prevalences were analyzed (Table 3 and Figure 2).

PCOS was the group with more obese women (34.5%), independently of the obese class considered. Interestingly, the majority of the women were normoweight in the NCAH (54.9%), IHA (54.5%), IH (61.2%), and control (73.5%) groups. The only exception was the PCOS group, with 44% of patients having a normal weight. Since all groups apart from PCOS had merely 25% or less patients in the obese subgroup, we performed an additional Kruskal–Wallis test only between PCOS Normoweight and PCOS Obese (Table 4). The results showed that PCOS Obese had significantly higher values in mFG, FT, and FAI (*p* < 0.001) and lower SHBG (*p* < 0.001) than PCOS Normoweight.

### 3.3. Correlation Analyses Results

To assess the association between mFG and other variables in each group, we calculated the Spearman Rho’s correlations (Tables S1–S5). Notably, we found weak but strongly significant correlations between mFG and FT (rho(278) = 0.23, *p* <  0.001), DHEAS (rho(282) = 0.13, *p* = 0.031), SHBG (rho(275) = −0.30, *p* < 0.001), FAI (rho(268) = 0.23, *p* <  0.001), FSH (rho(274) = −0.19, *p* = 0.001), and BMI (rho(280) = 0.30, *p* <  0.001) in the PCOS group. mFG was also weakly and negatively correlated with SHBG (rho(85) = −0.24, *p* = 0.025) in the IHA group. Moreover, we observed in the NCAH group two moderate positive correlations with mFG, FSH (rho(29) = 0.43, *p* = 0.016), and BMI (rho(29) = 0.42, *p* = 0.018).

Concerning the metabolic parameter in the PCOS group, BMI was positively correlated with FT (rho(250) = 0.34, *p* <  0.001) and FAI (rho(265) = 0.42, *p* <  0.001), and negatively correlated with SHBG (rho(272) = −0.48, *p* <  0.001). A similar association pattern for BMI was observed in the IHA group. NCAH also presented moderate significant correlations between BMI and SHBG (rho(28) = −0.41, *p* = 0.025) and FAI (rho(27) = 0.48, *p* =  0.009). In the IH group, we also identified significant although weak correlations of BMI with SHBG and FAI, but also with 17-OHP (rho(101) = 0.389, *p* <  0.001).

Interestingly, only in the PCOS group and the controls, strong positive correlations between TT and FAI (rho(270) = 0.64; rho(47) = 0.72, *p* <  0.001, respectively) were observed, whilst in the other groups, this correlation was just moderate. Several strong, positive, and highly significant correlations were found in the NCAH group. In particular, strong associations were found between FT and A4 (rho(29) = 0.78, *p* <  0.001 ), FT and 17-OHP (rho(29) = 0.63, *p* <  0.001), A4 and 17-OHP (rho(30) = 0.67, *p* <  0.001), and A4 and DHEAS (rho(30) = 0.66, *p* <  0.001). A strong correlation between SHBG and FAI was observed in all the groups, including the controls.

### 3.4. ROC Analyses

**Figure 3:**
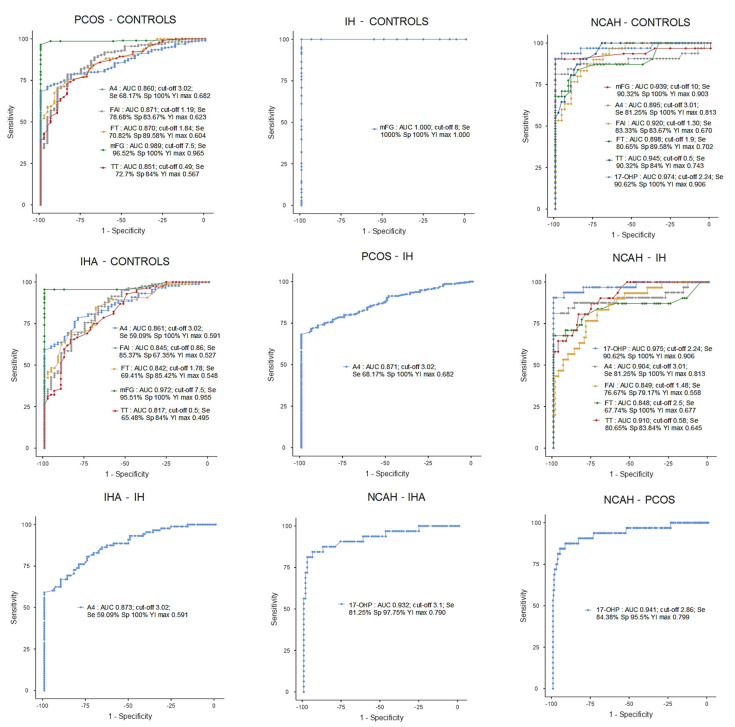
ROC curve results.

Several ROC analyses were performed to assess the area under the curve (AUC) of each variable between the groups and, therefore, measure its discrimination power for each model, in which a between-group comparison was considered (Figure 3).

In the PCOS–Controls model, mFG showed the best discrimination power (AUC: 0.989, *p* < 0.001). However, other variables such as TT (AUC: 0.851, *p* < 0.001), FT (AUC: 0.870, *p* < 0.001), A4 (AUC: 0.860, *p* < 0.001), and FAI (AUC: 0.871, *p* < 0.001) also presented good discrimination power.

The IHA–Controls model showed a similar pattern to the PCOS–Controls model.

None of the AUCs in the PCOS-NCAH and PCOS-IHA models were indicative of good or excellent discrimination power, except for 17-OHP, which had excellent discrimination power in the NCAH-PCOS model (AUC: 0.941, *p* < 0.001).

The highest AUC in both the PCOS-IH and IHA-IH models was A4 (AUC: 0.871, *p* < 0.001; AUC: 0.873, *p* < 0.001, respectively) with good discrimination power between the groups.

In the NCAH–Controls model, the variables with excellent discrimination power were mFG (AUC: 0.939, *p* < 0.001), TT (AUC: 0.945, *p* < 0.001), FAI (AUC: 0.920, *p* < 0.001), and, as expected, 17-OHP (AUC: 0.974, *p* < 0.001). Nonetheless, FT (AUC: 0.898, *p* < 0.001) and A4 (AUC: 0.895, *p* < 0.001) also showed good discrimination power.

The only variable with excellent discrimination power in the NCAH-IHA model was 17-OHP (AUC: 0.932, *p* < 0.001). In the NCAH-IH model, the highest AUC values were TT (AUC: 0.910, *p* < 0.001), A4 (AUC: 0.904, *p* < 0.001), and 17-OHP (AUC: 0.975, *p* < 0.001), with excellent discrimination power, and FT (AUC: 0.848, *p* < 0.001) and FAI (AUC: 0.849, *p* < 0.001), with only good discrimination power. Furthermore, a perfect model was found for specificity and sensitivity for the variable mFG in IH–Controls (Figure 3).

## 4. Discussion

This is the third study of large cohorts on the prevalence of PCOS in hyperandrogenic women after the landmark studies of Azziz [45] and Carmina [20].

This was a very comprehensive analysis of patients with hirsutism,—one of the largest series so completely studied. It sheds light on the different prevalences of the most frequent hyperandrogenic syndromes in a European population without the obesity levels that characterize studies from the USA. It demonstrates that besides PCOS, several other similar syndromes are also important and must be excluded, namely NCAH, idiopathic hirsutism and idiopathic hyperandrogenemia. Since the protocol was systematically applied to all patients, we were able to learn the relative contributions of adrenal and ovarian androgens to the different syndromes, calling attention, for instance, to the relative importance of adrenal androgen’s contribution to idiopathic hyperandrogenemia syndrome. Non-classic CAH, a disease which is very prevalent among hirsute women in Portugal, was demonstrated to be clinically similar to PCOS and to have even more elevated levels of androgens than PCOS. This points highlights the need to study 17OH progesterone secretion in every patient with hirsutism. We confirmed that insulin resistance is very frequent in PCOS as well as in other syndromes, and in spite of being clearly associated with obesity, it is also present significantly in lean hyperandrogenic patients. All these results will help personalize both analyses of etiologic factors as well as therapeutic interventions.

In this study, our primary objective was to determine the relative prevalence of PCOS, NCAH, IHA, and IH in a cohort of Portuguese hirsute women. We found a prevalence of 56.2% for PCOS, 6.2% for NCAH, 17.3% for IHA, and 20.2% for IH. These results are relatively comparable to the prevalence of PCOS of around 71% in European studies [5,10], NCAH of 3% to 5% [46], and IHA of 15.8% [20] assessed in other studies with hirsute women, but not for IH. Comparably, our results show a prevalence of IH two times higher than the one described by Escobar-Morreale et al. [5] (10%) and much more aligned with the one reported by Azziz et al. [47], who estimated an IH prevalence of 17% among hirsute women. However, it should be noted that prevalences depend on the population studied and on the diagnostic criteria used. For instance, a study determined that PCOS prevalence was between 6.1% and 15.3% depending on the diagnostic criteria used [48], while another study in a different population found a PCOS prevalence of only 2.2% [49].

PCOS patients showed higher levels than IH patients and the controls in several androgenic biomarkers. These results were also expected considering that most PCOS patients have biochemical hyperandrogenism [50] and that IH is characterized by normal serum androgen levels, despite its typical hirsute phenotype [23]. Indeed, the same rationale may also explain why PCOS patients showed lower SHBG only compared to both IH patients and the controls and no other hyperandrogenic conditions. After all, higher testosterone levels suppress SHBG in the liver, leaving FT and other androgens more available in the bloodstream [6,51,52], and together, these contribute to the progression of ovarian pathology, anovulation, and the phenotypic characteristics of PCOS [53]. On the contrary, the lack of influence of androgens on ovarian morphology and function in IHA patients is, in that sense, hard to understand.

Several studies found that lower levels of SHBG in PCOS patients specifically were negatively associated with obesity, particularly abdominal obesity [54,55,56]. In our study, PCOS was the group with both the lowest levels of SHBG and highest BMI, and the only group that presented a significant difference in BMI values compared to the controls.

PCOS was also the group with more obese and fewer normoweight patients. Obese PCOS patients had significantly higher values in mFG, FT, and FAI and lower SHBG than PCOS Normoweight patients.

Epidemiological data strongly support the association between obesity and PCOS, with studies indicating that 38–88% of women with PCOS are overweight or obese [57,58,59,60]. This led Carmina to suggest that obesity should be considered a new diagnostic criterion in PCOS [61]. Furthermore, according to a meta-analysis, obese women have 2.77 times the odds of developing PCOS compared to non-obese women [62]. Recent data indicate conflicting findings regarding hirsutism scores and BMI. Indeed, while some studies found that PCOS patients were more hirsute and obese than controls [63,64], others reported no such difference [41,43,65]. Selection bias may explain these inconsistencies, as patients from unselected populations often exhibit milder signs and symptoms compared to those in clinical settings [25]. For instance, a study in an unselected population found an obesity prevalence of 15% among PCOS patients diagnosed using the Rotterdam criteria, while PCOS prevalence among obese individuals was 30% [49].

The mean BMI of women with PCOS can also differ by country, reflecting national obesity rates [61]. In particular, in three different studies, the mean BMI levels were 29 kg/m^2^ in Italy [66], 31 kg/m^2^ in Germany [67], and 35.2 kg/m^2^ in the USA [68].

A recent meta-regression study found that mFG scores were positively related to testosterone and its precursors A4 and DHEAS, without finding any other significant relationships of the biochemical markers in PCOS assessed with mFG score [69]. Indeed, the pathogenesis of hirsutism is complex and depends on several factors, such as the idiosyncratic reaction of the pilosebaceous unit to androgens, increased levels of androgens, androgen receptor activity, and a possible deficit in the production of 5-α reductase and other key enzymes [70]. Specifically, 5-α reductase plays a key role in the biosynthesis and metabolism of androgens and their precursors, influencing the growth of sebaceous glands and hair follicles [71]. Nonetheless, the hair follicle response to the circulating levels of androgens varies, as evidenced by patients who show high levels of free or total testosterone without remarkable levels of hirsutism [5]. An observation study reported that TT did not correlate with mFG [72]. Conversely, a previous study observed an association between FAI and total testosterone in hyperandrogenism, with high precursor levels only in some patients [45]. Changes in hair growth have been positively associated with A4 levels and related to clinical manifestations of hirsutism in normoandrogenic women [73]. Moreover, the severity of hirsutism could differ based on the age of the patients [74], and possibly on genetic factors. Polymorphisms of the androgen receptor can influence the activity of the receptor [75]. Nevertheless, in a recent study [76], the association between single-nucleotide polymorphisms (SNPs) related to PCOS in Caucasian women was found not to be significantly associated with hirsutism, as assessed using mFG scores. These SNPs were also found to be significantly associated with PCOS in a cohort of Chinese women [77]. Despite our study not assessing the presence of these SNPs, our findings showed a significant difference between PCOS patients and healthy controls in both FT and TT and their precursors DHEAS and A4, and hirsutism was correlated to the circulating levels of androgens in PCOS patients, contrary to IH patients. A4 and TT also showed high discriminant power between the PCOS group, the control group, and the other patient groups. These results are consistent with previously published evidence. Hyperandrogenism and its clinical manifestations can be observed in PCOS patients.

Interestingly, in a recent study [78], the authors found a positive correlation between hirsutism score and FAI and a negative correlation with SHBG in both PCOS and NCAH patients. Our data presented similar results in PCOS women, but not in NCAH. Indeed, in our study, besides the PCOS group, no correlation between mFG and FAI was found any of the patient groups. Instead, we only found the same negative correlation in the IHA group. In this study, we found no significant differences in either hirsutism levels or FAI between PCOS and NCAH, consistent with the previous study [78]. Nonetheless, a recent meta-regression reported no significant associations of FG scores with FAI and SHBG in PCOS [69]. This discrepancy suggests that PCOS-associated hirsutism may depend on additional factors beyond circulating androgens, such as local androgen metabolism or receptor sensitivity.

As observed in PCOS, IH patients were younger and had higher hirsutism scores than the controls. Despite androgen levels not being significantly elevated, our results demonstrate increased FAI in IH patients compared to the controls, probably as a result of lower SHBG levels. This has already been reported in another study [75]. On the contrary, we found lower FAI in IH patients compared to PCOS and NCAH patients, despite SHBG not being significantly higher than in NCAH patients, like in PCOS patients. In these situations, lower FAI probably resulted from IH patients having reduced circulating androgens than NCAH patients, with lower total and free T, androstenedione, and DHEAS levels.

Patients with NCAH were more hirsute than the healthy controls. As confirmed by prior studies [20], this group had higher levels of A4, TT, and FT, but no difference in hirsutism when compared to the other patient groups. As corroborated by our results, due to significant clinical and hormonal similarities, only 17-OHP levels allow us to discriminate between PCOS and NCAH [79]. In addition, we also found that the 17-OHP values were able to discriminate between the NCAH and IHA groups. In addition, the hirsutism score does not correlate with genotype [80].

IH patients had a lower LH and LH/FSH ratio than PCOS, thus not demonstrating the hypothalamic–pituitary–ovarian (HPO) dysfunction observed in PCOS patients as a group. Indeed, HPO axis imbalance is a significant pathophysiology observation that more typically occurs in PCOS. Hypothalamic gonadotropin-releasing-hormone-secreting (GnRH) neurons play a role in PCOS via the altered regulation of LH synthesis through an abnormally elevated GnRH pulse, LH pulse frequency, and amplitude, further enhancing androgen synthesis in ovarian theca cells [81].

In what concerns IH, evidence points out that the circulating androgens are irrelevant. The skin can be conceived as an endocrine peripheral organ with all the enzymes needed to synthesize and catabolize androgens, using androgen precursors or even cutaneous cholesterol [82]. Testosterone in the skin results from converting the circulating DHEAS through the steroid sulphatase (STS), 3β-hydroxysteroid dehydrogenase (HSD), and 17β-HSD [83]. A recent study investigated the role played by skin local steroids, at sub-umbilical and arm levels, in women with IH compared to healthy controls [84]. They found increased mRNA expression of STS and type 2-17β-HSD, an enzyme usually present in the pilosebaceous unit. Despite this, increased mRNA expression was found in the skin samples from both body areas in women with IH.

The IHA group reported higher hirsutism scores than the controls. Compared to the IH and control groups, the IHA group showed significantly greater androgen levels, such as TT and FT; higher FAI values; and higher levels of testosterone’s adrenal precursors, A4 and DHEAS. Indeed, adrenal hyperandrogenism is common in patients with idiopathic hyperandrogenemia [85], even though the androgen source in idiopathic hyperandrogenemia is both ovarian and adrenal, like in PCOS. Furthermore, in a cohort of idiopathic hyperandrogenism patients, 48.3% presented elevated circulating DHEAS levels, suggesting that adrenal hyperandrogenism may be even more frequent in this mild androgen disorder than in PCOS [86] as shown in our data.

So, our study confirmed that there is a significant contribution of androgens to hirsutism in some of the analyzed syndromes but not in others, and that obesity’s influence is particularly prevalent only in PCOS. Furthermore, the present study provides a more comprehensive comparison by including hirsute women with other less common hyperandrogenic conditions, such as NCAH and IHA, and non-hyperandrogenic syndromes like IH. This approach is particularly significant given that few studies on IHA are currently published. Therefore, it adds further knowledge on the prevalence and biochemical profiles of these conditions within the Portuguese population. These findings contribute to a better understanding of the clinical variations within these syndromes, leading to more individualized and effective clinical management and treatment protocols for women with hirsutism.

Nonetheless, several limitations need to be acknowledged. Firstly, the sample sizes between patient groups are heterogeneous, even though the prevalences of each of the studied pathological conditions are significant. Moreover, although the sample may be representative of the Portuguese population, it is important to underline that it is not a multicentric or a multi-ethnic study, which might contribute more information to improve our knowledge of hirsutism syndromes in women and for future investigations on specific therapies.

## 5. Conclusions

This study aimed to describe the features of the various hirsutism syndromes in a cohort of Portuguese women, not only in order to better characterize them, but also to allow for a comparison to the situation in other ethnicities in the future.

Hirsutism is a complex condition that is more prevalent in Mediterranean, Middle Eastern, and South Asian ethnicities and is also influenced by skin type, since a positive correlation was found between hirsutism and Fitzpatrick classification, which scores skin type according to its susceptibility to sunburn and melanin production in response to sunlight.

Besides PCOS, hirsutism can be caused by non-classic congenital adrenal hyperplasia and idiopathic hyperandrogenism, which are less common hirsutism-related hyperandrogenic syndromes. While hirsutism is often an indicator of high circulating androgen levels resulting from ovarian or adrenal production, it can also be caused by other hormonal imbalances such as enhanced peripheral conversion of several androgen precursors to testosterone, and can even not be correlated to the levels of circulating androgens, as in idiopathic hirsutism. So, the presence of hirsutism does not differentiate these syndromes and only distinguishes them from cases of non-hirsute women.

Oligomenorrhea and polycystic ovarian morphology distinguish PCOS from idiopathic hyperandrogenism and idiopathic hirsutism.

NCAH demonstrated higher androgenic levels than PCOS, and the only way to discriminate these two syndromes is through 17OH-progesterone. DHEA-s is elevated in NCAH and PCOS but even more in idiopathic hyperandrogenism, a syndrome that seems to have a significant adrenal contribution to its pathogenesis.

Obesity only seems to contribute to the pathogenesis of PCOS, being associated with higher androgenic levels and lower SHBG.

## Figures and Tables

**Table 1:** The number of cases with levels above the upper limit of normality (and percentages).

		PCOS	NCAH	IHA	IH	Controls
mFG	N_Total_	287	31	89	103	50
	N_mFG>15.00_ (%)	141 (49.1%)	16 (51.6%)	36 (40.4%)	47 (45.6%)	-
TT	N_Total_	282	31	84	99	50
	N_TT>0.73_ (%)	109 (38.7%)	17 (54.8%)	21 (25.0%)	-	-
FT	N_Total_	257	31	85	77	48
	N_FT>2.50_ (%)	124 (48.2%)	19 (61.3%)	30 (35.3%)	-	-
A4	N_Total_	289	32	88	104	49
	N_A4>3.00_ (%)	197 (68.2%)	26 (81.3%)	52 (59.1%)	-	-
DHEAS	N_Total_	286	32	89	103	49
	N_DHEAS>4300.00_ (%)	33 (11.5%)	9 (28.1%)	10 (11.2%)	-	-
SHBG	N _Total_	279	31	87	101	49
	N_SHBG<32.4_ (%)	132 (47.3%)	14 (45.2%)	42 (48.3%)	30 (29.7%)	6 (12.2%)
FAI	N_Total_	272	30	82	96	49
	N_FAI>5.00_ (%)	22 (8.1%)	5 (16.7%)	3 (3.7%)	-	-
LH	N_Total_	278	32	83	98	49
	N_LH>12.6_ (%)	46 (16.5%)	4 (12.5%)	3 (3.6%)	2 (2.0%)	-
FSH	N_Total_	278	32	83	98	49
	N_FSH<3.5_ (%)	20 (7.2%)	5 (15.6%)	7 (8.4%)	10 (10.2%)	2 (4.1%)
LH-FSH ratio	N_Total_	278	32	83	98	49
	N_LH-FSH ratio>2.00_ (%)	62 (22.3%)	4 (12.5%)	8 (9.6%)	5 (5.1%)	-
17-OHP	N_Total_	289	32	89	104	49
	N_17-OHP>2.00_ (%)	63 (21.8%)	29 (90.6%)	32 (36.0%)	-	-
BMI	N_Total_	284	31	88	103	49
	N_BMI>30.00_ (%)	98 (34.5%)	2 (6.5%)	22 (25.0%)	17 (16.5%)	7 (14.3%)

**Table 2:** Between-group comparisons for each variable considered.

	Groups	$\tilde{x}$(± IQR)	PCOS	NCAH	IHA	IH	Normal
Age	PCOS	22.00 (±9.00)	–	0.531	0.853	0.761	<0.001 ****
	NCAH	19.50 (±8.25)	0.531	–	0.751	0.259	< 0.001 ****
	IHA	20.00 (±8.00)	0.853	0.751	–	0.448	<0.001 ****
	IH	22.50 (±12.00)	0.761	0.259	0.448	–	0.011 *
	Normal	28.00 (±11.25)	<0.001 ****	<0.001 ****	<0.001 ****	0.011 *	–
mFG	PCOS	15.00 (±6.50)	–	0.997	0.970	0.696	<0.001 ****
	NCAH	15.50 (±5.25)	0.997	–	0.928	0.738	<0.001 ****
	IHA	14.50 (±6.00)	0.970	0.928	–	0.738	<0.001 ****
	IH	14.50 (±4.25)	0.696	0.738	0.738	–	<0.001 ****
	Normal	5.25 (±2.38)	<0.001 ****	<0.001 ****	<0.001 ****	<0.001 ****	–
TT	PCOS	0.65 (±0.39)	–	0.020 *	0.084	<0.001 ****	<0.001 ****
	NCAH	0.81 (±0.54)	0.020 *	–	<0.001 ****	<0.001 ****	<0.001 ****
	IHA	0.57 (±0.31)	0.084	<0.001 ****	–	<0.001 ****	<0.001 ****
	IH	0.41 (±0.22)	<0.001 ****	<0.001 ****	<0.001 ****	–	0.175
	Normal	0.36 (±0.21)	<0.001 ****	<0.001 ****	<0.001 ****	0.175	–
FT	PCOS	2.50 (±1.70)	–	0.237	0.183	<0.001 ****	<0.001 ****
	NCAH	3.18 (±2.24)	0.237	–	0.017 *	<0.001 ****	<0.001 ****
	IHA	2.10 (±1.32)	0.183	0.017 *	–	<0.001 ****	<0.001 ****
	IH	1.50 (±0.55)	<0.001 ****	<0.001 ****	<0.001 ****	–	0.010 *
	Normal	1.30 (±0.79)	<0.001 ****	<0.001 ****	<0.001 ****	0.010 *	–
A4	PCOS	3.44 (±1.89)	–	0.009 **	0.643	<0.001 ****	<0.001 ****
	NCAH	4.90 (±4.15)	0.009 **	–	0.003 ***	<0.001 ****	<0.001 ****
	IHA	3.28 (±1.36)	0.643	0.003 ***	–	<0.001 ****	<0.001 ****
	IH	2.04 (±1.01)	<0.001 ****	<0.001 ****	<0.001 ****	–	0.911
	Normal	2.23 (±0.92)	<0.001 ****	<0.001 ****	<0.001 ****	0.911	–
DHEAS	PCOS	2455.00 (±1499.50)	–	0.255	0.007 **	0.156	<0.001 ****
	NCAH	2924.50 (±2217.50)	0.255	–	0.995	0.013 *	0.001 ***
	IHA	2980.00 (±1190.00)	0.007 **	0.995	–	<0.001 ****	<0.001 ****
	IH	2290.00 (±1263.50)	0.156	0.013 *	<0.001 ****	–	0.207
	Normal	1771.00 (±1380.00)	<0.001 ****	0.001 ***	<0.001 ****	0.207	–
SHBG	PCOS	33.50 (±26.70)	–	1.000	0.998	0.003 ***	<0.001 ****
	NCAH	37.40 (±30.35)	1.000	–	1.000	0.368	0.019 *
	IHA	33.80 (±25.90)	0.998	1.000	–	0.067	<0.001 ****
	IH	43.90 (±33.80)	0.003 ***	0.368	0.067	–	0.124
	Normal	52.00 (±26.10)	<0.001 ****	0.019 *	<0.001 ****	0.124	–
FAI	PCOS	1.91 (±1.72)	–	0.475	0.285	<0.001 ****	<0.001 ****
	NCAH	2.48 (±2.24)	0.475	–	0.056	<0.001 ****	<0.001 ****
	IHA	1.56 (±1.43)	0.285	0.056	–	<0.001 ****	<0.001 ****
	IH	0.92 (±0.75)	<0.001 ****	<0.001 ****	<0.001 ****	–	0.014 *
	Normal	0.66 (±0.61)	<0.001 ****	<0.001 ****	<0.001 ****	0.014 *	–
LH	PCOS	6.40 (±6.38)	–	0.056	<0.001 ****	<0.001 ****	0.027 *
	NCAH	4.75 (±4.70)	0.056	–	0.996	0.993	0.965
	IHA	4.50 (±3.30)	<0.001 ****	0.996	–	0.631	0.909
	IH	4.13 (±2.29)	<0.001 ****	0.993	0.631	–	0.175
	Normal	5.20 (±3.50)	0.027 *	0.965	0.909	0.175	–
FSH	PCOS	5.70 (±2.00)	–	0.492	0.999	0.989	0.058
	NCAH	4.95 (±2.10)	0.492	–	0.791	0.847	0.031 *
	IHA	5.70 (±2.59)	0.999	0.791	–	1.000	0.168
	IH	5.80 (±2.44)	0.989	0.847	1.000	–	0.135
	Normal	6.30 (±1.92)	0.058	0.031 *	0.168	0.135	–
LH-FSH ratio	PCOS	1.12 (±1.05)	–	0.098	<0.001 ****	<0.001 ****	<0.001 ****
	NCAH	0.86 (±1.10)	0.098	–	0.999	0.998	1.000
	IHA	0.80 (±0.54)	<0.001 ****	0.999	–	0.926	1.000
	IH	0.73 (±0.52)	<0.001 ****	0.998	0.926	–	0.983
	Normal	0.81 (±0.62)	<0.001 ****	1.000	1.000	0.983	–
17-OHP	PCOS	1.42 (±0.79)	–	<0.001 ****	0.694	<0.001 ****	<0.001 ****
	NCAH	7.15 (±8.48)	<0.001 ****	–	<0.001 ****	<0.001 ****	<0.001 ****
	IHA	1.50 (±1.17)	0.694	<0.001 ****	–	<0.001 ****	<0.001 ****
	IH	1.08 (±0.65)	<0.001 ****	<0.001 ****	<0.001 ****	–	1.000
	Normal	1.11 (±0.43)	<0.001 ****	<0.001 ****	<0.001 ****	1.000	–
BMI	PCOS	25.68 (±9.97)	–	0.489	0.774	0.053	0.003 ***
	NCAH	24.02 (±4.85)	0.489	–	0.941	0.999	0.417
	IHA	24.56 (±7.69)	0.774	0.941	–	0.764	0.060
	IH	23.62 (±5.71)	0.053	0.999	0.764	–	0.336
	Normal	22.15 (±4.65)	0.003 ***	0.417	0.060	0.336	–

Note: * *p* < 0.05 (uncorrected); ** *p* < 0.01 (uncorrected); *** *p* < 0.005 (corrected); **** *p* <0.001 (corrected).

**Table 3:** The number of subjects divided based on BMI results.

	Groups	PCOS N = 284 (51.2%)	NCAH N = 31 (5.6%)	IHA N = 88 (15.9%)	IH N = 103 (18.5%)	Normal N = 49 (8.8%)
BMI	Normoweight (<25)	n = 125 (44.0%) $\tilde{x}\;$= 21.8 (±2.87)	n = 17 (54.9%) $\tilde{x}\;$= 22.5 (±2.10)	n = 48 (54.5%) $\tilde{x}\;$= 22.4 (±2.88)	n = 63 (61.2%) $\tilde{x}\;$= 22.2 (±2.93)	n = 36 (73.5%) $\tilde{x}$ = 21.4 (±2.12)
	Overweight (≥25 and <30)	n = 61 (21.5%) $\tilde{x}\;$= 26.5 (±2.49)	n = 12 (38.7%) $\tilde{x}$ = 27.3 (±2.07)	n = 18 (20.5%) $\tilde{x}$ = 28.0 (±2.99)	n = 23 (22.3%) $\tilde{x}$ = 26.8 (±1.86)	n = 6 (12.2%) $\tilde{x}$ = 26.9 (±3.32)
	Obese Class 1 (≥30 and <35)	n = 54 (19.0%) $\tilde{x}$ = 31.9 (±1.95)	n = 1 (3.2%)	n = 12 (13.6%) $\tilde{x}$ = 32.7 (±2.93)	n = 9 (8.7%) $\tilde{x}$ = 31.5 (±1.17)	n = 5 (10.2%) $\tilde{x}$ = 32.5 (±1.09)
	Obese Class 2 (≥35 and <40)	n = 28 (9.9%) $\tilde{x}$ = 37.5 (±2.74)	n = 0	n = 7 (8.0%) $\tilde{x}$ = 36.5 (±1.27)	n = 5 (4.9%) $\tilde{x}$ = 37.6 (±1.69)	n = 2 (4.1%) $\tilde{x}$ = 37.3 (±0.40)
	Obese Class 3 (≥ 40)	n = 16 (5.6%) $\tilde{x}$ = 42.8 (± 3.43)	n = 1 (3.2%)	n = 3 (3.4%) $\tilde{x}$ = 41.5 (± 2.60)	n = 3 (2.9%) $\tilde{x}$ = 40.4 (± 0.63)	n = 0

**Table 4:** PCOS Normoweight and PCOS Obese comparison.

	Group	N	$\tilde{x}$ (±IQR)	*p*-Value
mFG	PCOS Normoweight	123	14.50 (±5.50)	<0.001 ****
	PCOS Obese	98	17.25 (±7.38)	
TT	PCOS Normoweight	123	0.64 (±0.33)	0.072
	PCOS Obese	94	0.68 (±0.39)	
FT	PCOS Normoweight	109	2.00 (±1.28)	<0.001 ****
	PCOS Obese	93	2.80 (±1.84)	
A4	PCOS Normoweight	125	3.38 (±1.82)	0.161
	PCOS Obese	98	3.66 (±1.98)	
DHEAS	PCOS Normoweight	124	2445.00 (±1275.00)	0.474
	PCOS Obese	97	2600.00 (±1670.00)	
SHBG	PCOS Normoweight	117	42.50 (±30.00)	0.001 ****
	PCOS Obese	98	23.35 (±15.03)	
FAI	PCOS Normoweight	115	1.61 (±1.22)	<0.001 ****
	PCOS Obese	94	2.75 (±2.36)	
LH	PCOS Normoweight	122	6.40 (±6.28)	0.274
	PCOS Obese	94	5.54 (±7.65)	
FSH	PCOS Normoweight	122	5.90 (±1.68)	0.684
	PCOS Obese	94	5.61 (±1.68)	
LH-FSH ratio	PCOS Normoweight	122	1.05 (±0.91)	0.388
	PCOS Obese	94	1.13 (±1.24)	
17-OHP	PCOS Normoweight	125	1.43 (±0.77)	0.570
	PCOS Obese	98	1.41 (±0.82)	

Note: **** *p* < 0.001 (corrected).

## Data Availability

The data are available from the corresponding author on reasonable request.

## Supplementary Materials

The following supporting information can be downloaded at: https://www.mdpi.com/article/10.3390/jcm14030673/s1, Table S1: Correlation analysis results for PCOS group; Table S2: Correlation analysis results for NCAH group; Table S3: Correlation analysis results for IHA group; Table S4: Correlation analysis results for IH group; Table S5: Correlation analysis results for control group.

## Abbreviations

PCOS	Polycystic ovary syndrome
PCOM	Polycystic ovarian morphology
CAH	Congenital Adrenal Hyperplasia
NCAH	Non-classic congenital adrenal hyperplasia
IHA	Idiopathic hyperandrogenemia
IH	Idiopathic hirsutism
FST	Fitzpatrick skin type
BMI	Body mass index
mFG	Modified Ferriman–Gallwey
T	Testosterone
DHT	Dihydrotestosterone
AR	Androgen receptor
TT	Total testosterone
FT	Free testosterone
A4	Androstenedione
DHEAS	Dehydroepiandrosterone sulfate
SHBG	Sex hormone-binding globulin
ACTH	Adrenocorticotropic hormone
LH	Luteinizing hormone
FSH	Follicle-stimulating hormone
17-OHP	17-Hydroxyprogesterone basal
FAI	Free androgen index
$\tilde{x}$	Median
IQR	Interquartile range
DSCF	Dwass–Steel–Critchlow–Fligner
ROC	Receiver operating characteristic
AUC	Area under the curve
SE	Sensibility
SP	Specificity
YI	Youden index
SNP	Single-nucleotide polymorphisms
GnRH	Gonadotropin-releasing hormone
STS	Steroid sulphatase
HDS	Hydroxysteroid dehydrogenase
mRNA	Messenger ribonucleic acid
