# Supplementary material for: Androgens and Hirsutism in a Large Cohort of Portuguese Women
Source: J Clin Med. 2025 Jan 21;14(3):673. doi: 10.3390/jcm14030673 (PMC11818050; doi:10.3390/jcm14030673)
Supplement: Supplementary file 1 [file jcm-14-00673-s001.zip › jcm-3372398-supplementary.pdf]

Journal of
*Clinical Medicine*

MDPI

# The Association between Androgens and Hirsutism in a Portuguese cohort of women – *Supplementary Materials*

**Table S1.** Correlation analyses results for PCOS Group

| | **mFG** | **TT** | **FT** | **A4** | **DHEAS** | **SHBG** | **FAI** | **LH** | **FSH** | **LH-FSH ratio** | **17-OHP** | **BMI** |
|---|---|---|---|---|---|---|---|---|---|---|---|---|
| mFG | — | | | | | | | | | | | |
| TT | -0.005 | — | | | | | | | | | | |
| FT | 0.227 *** | 0.389 *** | — | | | | | | | | | |
| A4 | -0.011 | 0.410 *** | 0.246 *** | — | | | | | | | | |
| DHEAS | 0.128 * | 0.171 ** | 0.217 *** | 0.246 *** | — | | | | | | | |
| SHBG | -0.301 *** | -0.021 | -0.324 *** | -0.068 | -0.192 ** | — | | | | | | |
| FAI | 0.233 *** | 0.637 *** | 0.478 *** | 0.310 *** | 0.245 *** | -0.758 *** | — | | | | | |
| LH | -0.073 | 0.252 *** | 0.128 * | 0.312 *** | -0.069 | 0.102 | 0.077 | — | | | | |
| FSH | -0.194 ** | -0.137 * | -0.075 | 0.044 | -0.079 | 0.029 | -0.120 | 0.265 *** | — | | | |
| LH-FSH ratio | 0.012 | 0.304 *** | 0.184 ** | 0.302 *** | -0.018 | 0.071 | 0.135 * | 0.864 *** | -0.205 *** | — | | |
| 17-OHP | 0.017 | 0.238 *** | 0.194 ** | 0.457 *** | 0.250 *** | -0.097 | 0.217 *** | 0.216 *** | -0.054 | 0.266 *** | — | |
| BMI | 0.297 *** | 0.109 | 0.341 *** | 0.077 | 0.058 | -0.478 *** | 0.423 *** | -0.065 | 0.006 | -0.063 | -0.035 | — |

Note. * $p < .05$, ** $p < .01$, *** $p < .001$

**Table S2.** Correlation analyses results for NCAH Group

| | mFG | TT | FT | A4 | DHEAS | SHBG | FAI | LH | FSH | LH-FSH ratio | 17-OHP | BMI |
|---|---|---|---|---|---|---|---|---|---|---|---|---|
| mFG | — | | | | | | | | | | | |
| TT | 0.131 | — | | | | | | | | | | |
| FT | -0.277 | 0.525 ** | — | | | | | | | | | |
| A4 | -0.221 | 0.557 ** | 0.779 *** | — | | | | | | | | |
| DHEAS | -0.010 | 0.444 * | 0.564 *** | 0.664 *** | — | | | | | | | |
| SHBG | -0.095 | 0.047 | -0.092 | 0.034 | -0.124 | — | | | | | | |
| FAI | 0.236 | 0.553 ** | 0.393 * | 0.274 | 0.353 | -0.794 *** | — | | | | | |
| LH | 0.092 | -0.040 | -0.134 | 0.031 | -0.173 | -0.262 | 0.225 | — | | | | |
| FSH | 0.430 * | 0.062 | 0.044 | 0.265 | 0.208 | -0.418 * | 0.405 * | 0.305 | — | | | |
| LH-FSH ratio | -0.135 | -0.034 | -0.073 | 0.044 | -0.168 | -0.037 | 0.019 | 0.828 *** | -0.108 | — | | |
| 17-OHP | -0.048 | 0.464 ** | 0.626 *** | 0.673 *** | 0.413 * | 0.230 | 0.051 | -0.207 | 0.351 * | -0.249 | — | |
| BMI | 0.421 * | 0.243 | 0.067 | -0.023 | 0.152 | -0.409 * | 0.482 ** | 0.069 | 0.153 | -0.012 | -0.153 | — |

Note. * $p < .05$, ** $p < .01$, *** $p < .001$

**Table S3.** Correlation analyses results for IHA Group

| | mFG | TT | FT | A4 | DHEAS | SHBG | FAI | LH | FSH | LH-FSH ratio | 17-OHP | BMI |
|---|---|---|---|---|---|---|---|---|---|---|---|---|
| mFG | — | | | | | | | | | | | |
| TT | -0.056 | — | | | | | | | | | | |
| FT | 0.007 | -0.099 | — | | | | | | | | | |
| A4 | -0.075 | -0.039 | -0.012 | — | | | | | | | | |
| DHEAS | 0.115 | 0.134 | 0.057 | 0.104 | — | | | | | | | |
| SHBG | -0.241 * | 0.042 | -0.283 ** | -0.014 | -0.199 | — | | | | | | |
| FAI | 0.132 | 0.504 *** | 0.202 | -0.016 | 0.218 * | -0.822 *** | — | | | | | |
| LH | 0.043 | -0.151 | 0.099 | 0.070 | -0.033 | 0.281 * | -0.332 ** | — | | | | |
| FSH | 0.083 | -0.259 * | -0.075 | 0.169 | -0.216 * | -0.061 | -0.109 | 0.156 | — | | | |
| LH-FSH ratio | -0.021 | 0.020 | 0.108 | -0.042 | 0.098 | 0.306 ** | -0.251 * | 0.774 *** | -0.447 *** | — | | |
| 17-OHP | -0.062 | -0.077 | -0.050 | 0.042 | 0.273 ** | 0.069 | -0.140 | 0.100 | 0.020 | 0.139 | — | |
| BMI | 0.057 | -0.114 | 0.338 ** | -0.076 | 0.028 | -0.595 *** | 0.453 *** | -0.179 | 0.021 | -0.177 | -0.077 | — |

Note. * p < .05, ** p < .01, *** p < .001

**Table S4.** Correlation analyses results for IH Group

| | mFG | TT | FT | A4 | DHEAS | SHBG | FAI | LH | FSH | LH-FSH ratio | 17-OHP | BMI |
|---|---|---|---|---|---|---|---|---|---|---|---|---|
| mFG | — | | | | | | | | | | | |
| TT | 0.018 | — | | | | | | | | | | |
| FT | 0.020 | 0.232 * | — | | | | | | | | | |
| A4 | -0.022 | 0.221 * | 0.215 | — | | | | | | | | |
| DHEAS | -0.043 | 0.361 *** | 0.405 *** | 0.212 * | — | | | | | | | |
| SHBG | 0.040 | 0.140 | 0.036 | -0.093 | -0.116 | — | | | | | | |
| FAI | 0.048 | 0.485 *** | 0.154 | 0.198 | 0.369 *** | -0.771 *** | — | | | | | |
| LH | 0.003 | 0.195 | 0.073 | 0.298 ** | 0.088 | 0.165 | -0.021 | — | | | | |
| FSH | -0.102 | -0.036 | 0.111 | 0.110 | 0.035 | 0.038 | -0.055 | 0.174 | — | | | |
| LH-FSH ratio | 0.045 | 0.207 * | -0.032 | 0.097 | 0.018 | 0.165 | -0.029 | 0.646 *** | -0.583 *** | — | | |
| 17-OHP | 0.135 | 0.014 | 0.139 | 0.395 *** | 0.171 | -0.026 | 0.046 | 0.158 | 0.137 | -0.050 | — | |
| BMI | 0.035 | 0.066 | -0.110 | -0.193 | -0.010 | -0.315 ** | 0.321 ** | -0.159 | -0.077 | -0.069 | -0.389 *** | — |

Note. * p < .05, ** p < .01, *** p < .001

**Table S5.** Correlation analyses results for Control Group

| | **mFG** | **TT** | **FT** | **A4** | **DHEAS** | **SHBG** | **FAI** | **LH** | **FSH** | **LH-FSH ratio** | **17-OHP** | **BMI** |
|---|---|---|---|---|---|---|---|---|---|---|---|---|
| mFG | — | | | | | | | | | | | |
| TT | -0.150 | — | | | | | | | | | | |
| FT | -0.116 | 0.261 | — | | | | | | | | | |
| A4 | -0.145 | 0.279 | -0.006 | — | | | | | | | | |
| DHEAS | 0.001 | 0.314 * | 0.352 * | 0.061 | — | | | | | | | |
| SHBG | -0.199 | 0.006 | -0.178 | 0.231 | -0.385 ** | — | | | | | | |
| FAI | 0.059 | 0.723 *** | 0.330 * | 0.115 | 0.437 ** | -0.609 *** | — | | | | | |
| LH | -0.050 | 0.050 | -0.143 | -0.045 | -0.020 | 0.027 | -0.016 | — | | | | |
| FSH | 0.149 | -0.192 | -0.196 | -0.055 | -0.152 | 0.059 | -0.196 | 0.029 | — | | | |
| LH-FSH ratio | -0.130 | 0.098 | -0.028 | -0.005 | 0.044 | 0.001 | 0.051 | 0.861 *** | -0.411 ** | — | | |
| 17-OHP | -0.055 | -0.158 | 0.104 | 0.215 | 0.335 * | -0.087 | -0.117 | -0.017 | -0.041 | 0.058 | — | |
| BMI | 0.021 | 0.164 | 0.237 | 0.031 | -0.033 | -0.027 | 0.112 | 0.239 | 0.224 | 0.102 | -0.229 | — |

Note. * $p < .05$, ** $p < .01$, *** $p < .001$
